# The potential protective roles of Astragalus polysaccharides against toxicity of polystyrene nanoplastics on mice sperm exposed

**DOI:** 10.3389/fvets.2026.1924767

**Published:** 2026-08-31

**Authors:** Qimeng Hu, Yuxin Xie, Jiaying Shi

**Affiliations:** Hebei Key Laboratory of Animal Diversity, Institute of Animal Diversity and Ecological Restoration, College of Life Sciences, Langfang Normal University, Langfang, Hebei, China

**Keywords:** Astragalus polysaccharides, mouse, polystyrene nanoplastics, sperm, tyrosine phosphorylation

## Abstract

**Introduction:**

Polystyrene nanoparticles (PS-NPs) damage the male reproductive system, although the potential impacts on mammalian reproductive health remain unclear. The aim of this study was to determine the protective effect of *Astragalus* polysaccharides (APS) on testicular and epididymal functions in a mouse against PS-NPs as environmental pollutants.

**Methods:**

Male mice were randomly assigned to three groups (n = 6 each): control, PS-NPs at 50 mg/kg/day (P group), and PS-NPs at 50 mg/kg/day plus APS at 100 mg/kg/day (P+A group), aimed to elucidate the molecular mechanisms of PS-NPs exposure on sperm and the protective effects of APS. Testicular microstructure and function, serum testosterone level, enrichment of PS-NPs in the epididymis, sperm morphology and motility, concentration of zinc ions, the CuZn-superoxide dismutase activity (SOD) activity, total antioxidant capacity (T-AOC), malondialdehyde content (MDA) in seminal plasma, and protein tyrosine phosphorylation of non-/capacitated sperm were evaluated.

**Results:**

Exposure to PS-NPs significantly decreased the percentage of normal seminiferous tubules and serum testosterone levels (*p* < 0.05). The seminal plasma Zn^2+^ levels, CuZn-SOD activity, T-AOC, and sperm quality (density, motility, normal morphology) significantly reduced, with the increased the enrichment of PS-NPs in the epididymis and MDA levels of sperm on PS-NPs treatment group (*p* < 0.05). Exposure to PS-NPs induced the 32 ~ 35KDa protein tyrosine phosphorylation of non-capacitated sperm (*p* < 0.05). The protein tyrosine phosphorylation of capacitated sperm exposure by PS-NPs were decreased compare with control group (*P* < 0.05), which located in sperm flagella. After APS treatment, the functional impairments caused by PS-NPs were alleviated, and there was no significant difference in related indicators compared to the physiological group (*p* > 0.05).

**Discussion:**

APS relieved oxidative damage to sperm, enhanced antioxidant capacity, and protected against testicular and epididymal damage caused by exposure to PS-NPs. APS regulated protein tyrosine phosphorylation to prevented the of “capacitation-like” phenomenon of non-capacitated sperm caused by PS-NPs. Meanwhile, APS alleviated the decrease in proteins tyrosine phosphorylation of capacitated sperm exposed by PS-NPs. These results indicate that APS could be used to ameliorate sperm quality induced by environmental toxins.

## 1 Introduction

As a newly emerging anthropogenic pollutant, exposure of the general population to nanoplastics (NPs) continues to increase and therefore the potential impacts on human health have become an emerging field of research. NPs, which are plastic particles ranging in size from 0.001 to 0.1 μm, can permeate through biofilms and have a relatively large surface area with the ability to adsorb other environmental pollutants, thereby increasing the toxicity. The major NPs present in the environment include polystyrene (PS), polyvinyl chloride, polypropylene, and polyethylene (1). PS is commonly used for food delivery packaging, home insulation, and cargo transportation. Due to commercial availability, multifunctionality, and difficulty in degradation, PS is often selected as a representative NP for studies of persistent pollutants (2). In addition, PS particles can be easily synthesized in various sizes, as ideal model particles for laboratory studies of environmental pollutants (3). Reportedly, humans consume approximately 2.4–700 mg of plastic pellets daily (4).

The large specific-surface area of PS-NPs facilitates the adsorption of oxidative by-products and hazardous substances, thus amplifying their toxic effects (5). Multiple related studies have confirmed that exposure to PS-NPs leads to the production of large amounts of reactive oxygen species (ROS) in cells, thereby triggering oxidative stress responses. PS-NPs can disrupt cellular oxidative balance and cause cell death by binding and consuming intracellular antioxidants.

*Astragalus* polysaccharides (APS) are the main bioactive components of *Astragalus membranaceus*. APS can alleviate mitochondrial damage by clearing excessive ROS from cells (6). The active ingredients of herbs used in traditional Chinese medicine have unique advantages in the field of toxicology due to their multi-component, multi-target, and multi-pathway mechanisms (7). Research has shown that APS can improve the sperm quality of rams, thereby enhancing reproductive performance (8). In addition, APS was reported to alleviate bufalin-induced oxidative damage to mouse testes through mechanisms involving the blood-testis barrier repair (6).

Although the global impact has been established, there were currently no specific or effective interventions to mitigate the reproductive toxicity caused by PS-NPs. Past research on the impact of accumulated PS-NPs on male reproductive physiology has mainly focused on the toxic effects on reproductive organs and sperm production, but the mechanisms of action during the sperm capacitation remains unclear. Therefore, the aims of this study were to investigate the effects of PS-NPs on sperm non-/capacitation and testicular and epididymal functions in a mouse model, and the physiological protective mechanism of APS.

## 2 Methods and materials

### 2.1 Chemicals

The Astragalus polysaccharide used in this study was purchased from Yuanye BioTechnology (Shanghai yuanye Bio-Technology Co., Ltd., Shanghai, China; S27816), the purity of this APS preparation is approximately 60%.

Polystyrene nanoplastics (50 nm, −19.87 mV) and fluorescent polystyrene NPs with a size of 50 nm were purchased from WUXI RIGOR BIO-TECH CO., LTD (Wuxi, China).

### 2.2 Experimental animals and sperm collection

Adult ICR male mice (*n* = 30) weighing 25 ~ 30 g purchased from Sipeifu Biotechnology Co., Ltd. were selected for the experiment. After 1 week of acclimation period, they were randomly divided into three groups (*n* = 6). According to the experimental design, the treated groups of mice were orally administered 0.1 ml of sterile ddH_2_O containing PS-NPs and/or APS once a day. The control group of mice were orally administered 0.1 ml of sterile ddH_2_O. After 10 days of exposure, testes, epididymis, sperm, and blood were collected. The epididymis were placed in 1ml of non-capacitation culture medium, then cut the epididymis with scissors, and incubate at 37 °C for 10 min. The sperm swim out and the supernatant were collected for use.

To analysis enrichment of PS-NPs in the epididymis, used fluorescent PS-NPs at 50 mg/kg/day group and fluorescent PS-NPs at 50 mg/kg/day with APS at 100 mg/kg/day group (*n* = 6). After 3 days of gavage, PS-NPs fluorescence enrichment of the mouse epididymis was analyzed. All animal procedures conducted in this study comply with the National Research Council Laboratory Care and Use Guidelines for animals, and the proposal has been approved by the Academic Ethics and Science Ethics Committee of Langfang Normal University (2026KYLL1).

### 2.3 Study design and media

Mouse models were orally exposed to control group (Con), 50 mg/kg/day PS-NP group (P), 50 mg/kg/day PS-NPs and 100 mg/kg/day APS group (P+A). The detection indicators included: testicular function analysis (serum testosterone concentration and testicular histology), epididymal function analysis (epididymal fluorescence PS-NPs enrichment detection, sperm density, motility and deformity rate), seminal plasma physiological indicators (Zn^2+^, CuZn-SOD, T-AOC, and MDA content), and analysis of capacitated-dependent processes (protein tyrosine phosphorylation).

The non-capacitated incubation medium for mouse sperm included NaCl (100 mm), KCl (4.4 mM), KH_2_PO_4_ (1.2 mm), MgSO_4_ (1.2 mmol), Glucose (5.4 mM), calcium lactate (2.4 mm), HEPES (20 mm), and sodium pyruvate (0.8 mm). The capacitated incubation medium containing 20 mm NaHCO_3_ and 5 mg/ml BSA (bovine serum albumin), on the basis of non-capacitated incubation medium. In all cases, the medium were adjusted to pH 7.3 before used and keep the medium in a 37 °C bath. During incubation, the tubes were shaken gently every quarter of an hour to prevent precipitation.

### 2.4 Analysis of sperm morphology and motility

Sperm morphology was analyzed using a rapid sperm staining kit (Nanjing Jiancheng Bioengineering Institute). A semen suspension was prepared, spread evenly on a glass slide. The samples were air-dried naturally, and fixed with 95% ethanol for 3 min (9). The slide was hydrated for 2 min. The samples were stained with nuclear dye solution for 2 min and rinsed with water. The color enhancer solution was added and mixed for approximately 5 s, followed by washing with the washing solution. The acrosomal region of the sperm stained light purple, the post-acrosomal region stained dark purple, and the sperm flagellum stained pale red. 200 spermatozoa were counted for each sample.

Sperm motility was observed in a sperm counting chamber (MAILANG, China) prewarmed to 37 °C under a light microscope (CX21; Olympus Corporation). Sperm motility was calculated as the number of progressive sperm/200 × 100%.

### 2.5 Testicular histology

Testes were collected from mice in each group washed with PBS solution. The excess water was removed with absorbent paper. Histological features of the testes were evaluated as previously described (10). Briefly, the testes were fixed, dehydrated and cleared. The samples were embedded in paraffin wax, cut into 5-μm-thick sections, and mounted onto glass slides using a microtome (LEICA RM2125RT). The sections were stained with hematoxylin and eosin (HE) following standard protocols for morphological observation. The stained tissue sections were examined under an optical microscope (Olympus BX53; Olympus Optical Co, Ltd, Tokyo, Japan). The seminiferous tubules were evaluated according to Johnsen's testicular biopsy score.

### 2.6 Enrichment of PS-NPs in the epididymis

The PS-NPs (100 μl of 10 mg/ml) (Reg Biotech Co., Ltd.) with red fluorescent groups and 100 μl of ddH_2_O (negative control) to mice by gavage. On the third day after gavage, the epididymis were collected from mice stored in 4% paraformaldehyde. The sample was made into ultra-thin sections and observed under a fluorescence microscope to detect the accumulation of red fluorescent PS-NPs in the epididymis.

### 2.7 Serum testosterone testing

Serum testosterone levels in each group were measured using an enzyme linked immunosorbent assay kit purchased from Nanjing Jiancheng Bioengineering Institute. Testosterone concentrations were determined via enzyme-linked immunosorbent assay in strict accordance with the manufacturer's kit instructions. Absorbance values were detected with a microplate reader (Bio-Rad Laboratories) at a wavelength of 450 nm.

### 2.8 Concentration of zinc ions in seminal plasma

Semen zinc ion concentrations were detected using the semen zinc quantification kit (Huakang Biomedical Holdings Co., Ltd.). All operations were performed in accordance with the kit instructions, and absorbance was measured with a microplate reader (Bio-Rad Laboratories) at a wavelength of 490 nm.

### 2.9 Detection of CuZn-SOD activity in semen

CuZn-SOD activity was determined using a CuZn-SOD detection kit (Nanjing Jiancheng Bioengineering Institute, China) (11). Semen samples were washed three times with PBS. The sperm samples were mixed with the kit buffer, vortexed for 1 min and then centrifuged (12,000 × g, 5 min) to remove debris. The CuZn-SOD activity of samples from each group were measured with a spectrophotometer (UNICO UV-2102PCS) at a wavelength of 550 nm.

### 2.10 Total antioxidant capacity activity detection

T-AOC activity in semen was measured with the T-AOC detection kit (Nanjing Jiancheng Bioengineering Institute) (12). An aliquot of 1 ml of each specific sperm sample was washed and resuspended in ddH_2_O. Sperm were sonicated (four cycles at 20 kHz, 750 W, at 40% for 3 s, with a 5-s interval between cycles) and the suspended sperm were lysed on ice and then mixed with reaction buffer. Finally, T-AOC levels in each sample were measured with a spectrophotometer (UNICO UV-2102PCS) at a wavelength of 520 nm.

### 2.11 Detection of malondialdehyde content in semen

Malondialdehyde levels were detected using a malondialdehyde detection kit (Nanjing Jiancheng Bioengineering Institute, Jiangsu Province) (12). All experimental operations were conducted in accordance with the kit instructions. Briefly, sperm samples were washed three times with PBS, mixed with buffer solution, and then subjected to sonication. After centrifugation at 12,000 × g for 10 min, the supernatant was transferred into a new test tube, blended with reaction buffer, and vortexed thoroughly. The reaction mixture was incubated in a water bath at 95 °C for 40 min. The absorbance was determined with a spectrophotometer (UNICO UV-2102PCS) at a wavelength of 532 nm.

### 2.12 Evaluation of protein tyrosine phosphorylation of sperm

#### 2.12.1 Western blot

Protein samples were separated via 12% SDS-PAGE and transferred from the gel to a polyvinylidene fluoride membrane (0.45 μM) using an electrophoresis transfer tank at 10 V for 12 h. The polyvinylidene fluoride membrane was equilibrated with tris-buffered saline with Tween-20 at 4 °C for 5 min, followed by blocking for 1 h. The membrane was incubated at 4 °C for 4 h with anti-tyrosine phosphorylation antibody (dilution 1:10,000, clone 4G10, Cat# 05-321, EMD Millipore Corporation, Billerica, MA, USA) or the internal reference β-tubulin antibody (dilution 1:5,000, K200059M, Beijing Solarbio Science & Technology Co., Ltd., Beijing, China). After three times washed with tris-buffered saline with Tween-20, the membrane was incubated with the corresponding HRP-labeled Goat AntiMouse IgG (H+L) (dilution 1: 10,000, A0216, Beyotime Institute of Biotechnology) for 3 h. The membrane was treated with chemiluminescence reagent, and protein bands were visualized. Images were captured using a Bio-Rad gel imaging system.

### 2.13 Immunofluorescence staining

The formaldehyde solution (10%) was added to sperm samples from each group for fixation (4 °C, 12 h) (12). The fixed sperm samples were washed three times with PBS. A 60 μl aliquot of sperm sample was evenly spread onto a glass slide and air-dried at room temperature. The samples were then fixed twice with 3.7% formaldehyde solution for 20 min each time. The samples were washed three times with washing solution (PBS containing 0.1% Tween-20) for 3 min each time and permeabilized with permeabilization solution (PBS containing 0.5% Triton X-100) for 5 min. Subsequently, the samples were blocked with blocking solution (washing solution containing 1% BSA) for 2 h. The samples were incubated overnight with primary antibody (1:5,000, clone 4G10, Cat# 05-321, EMD Millipore Corporation, Billerica, MA, USA) diluted in blocking solution, followed by incubation with Alexa 488-conjugated anti-mouse antibody (1:1,000, A0428, Beyotime Institute of Biotechnology) for 2 h. The samples were washed three times with washing solution between each step. Antifade solution was added onto the samples, which were then covered with coverslips. Finally, the samples were observed and photographed under a fluorescence microscope at 400 × magnification (OLYMPUS DP73).

### 2.14 Statistical analysis

The data were presented as mean ± standard deviation. Significance of differences between the groups were assessed by one-way analysis of variance and Duncan's multiple range tests using SPSS 20.0 software. Fluorescence enrichment analysis of PS-NPs in the epididymis was performed using independent sample t-test with SPSS 20.0 software. The *p*-Values less than 0.05 were considered to indicate statistical significance.

## 3 Results

### 3.1 Testicular HE staining

**Figure 1:**
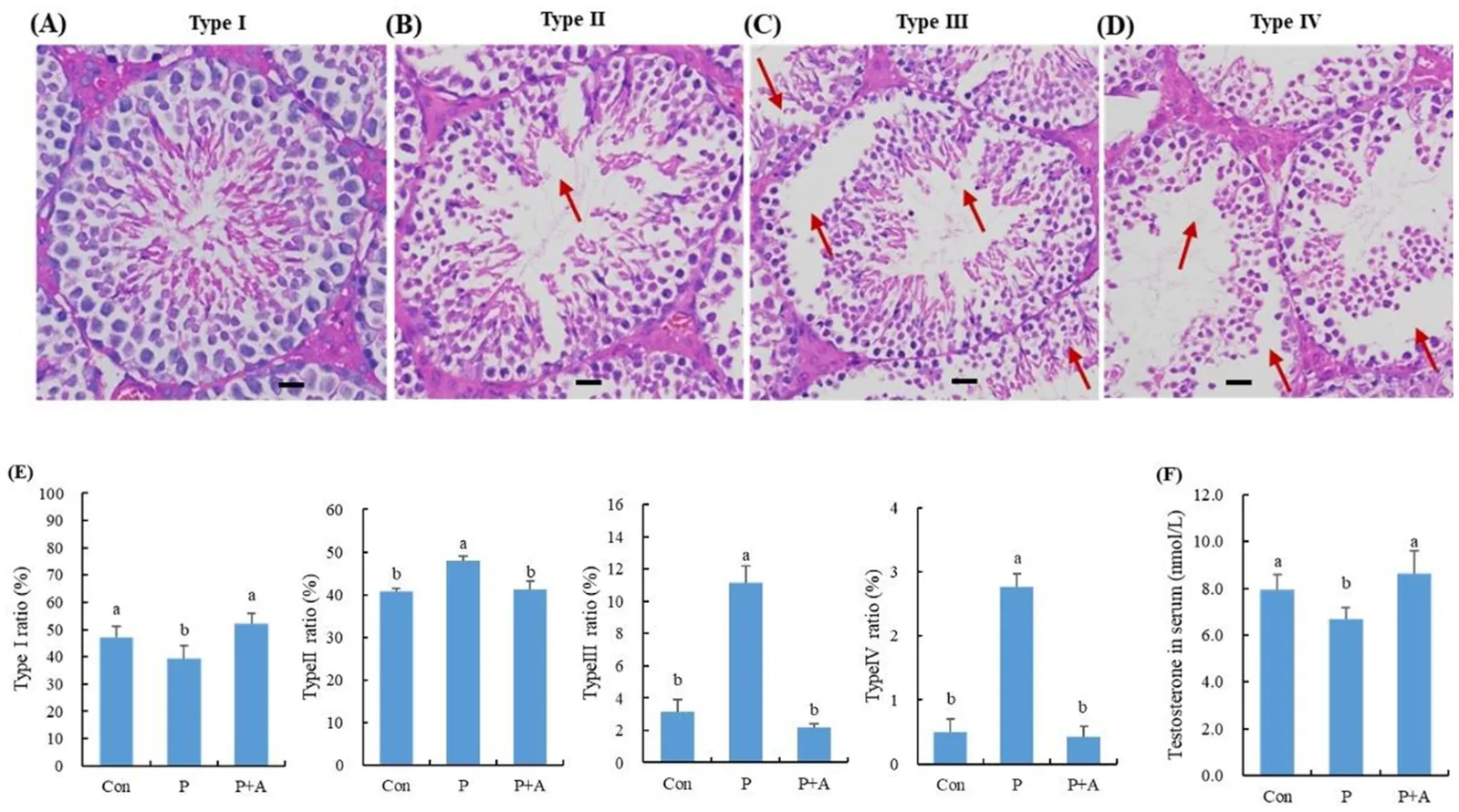
Effect of APS on the microstructure and function of testes in mice exposed to PS-NPs. **(A–D)** Four representative histopathological images of seminiferous tubules, Bar = 50 μm. **(E)** Type I-IV ratio. Arrows indicate loose arrangement of germ cells and cavities in the seminiferous tubules. **(F)** The effect of PS-NPs/APS on serum testosterone levels. Data are presented as the mean ± standard deviation (*n* = 6; *p < 0.05). Con: Control group; P group: PS-NPs at 50 mg/kg/day; P+A group: PS-NPs at 50 mg/kg/day and APS at 100 mg/kg/day. The number of biological replicates was 6 mice.

Based on the histopathological images of seminiferous tubules, the tubules were classified into four types. Type I represented normal seminiferous tubules with intact and smooth boundaries, neatly and densely arranged germ cells, and abundant sperm in the lumen (Figure 1A). Type II denoted abnormal seminiferous tubules containing enlarged vacuolar cavities, loosely arranged germ cells, and reduced sperm number in the lumen (Figure 1B). Type III exhibited more loosely organized germ cells and a greater number of vacuolar cavities compared with Type II (Figures 1B, C). Type IV showed disrupted boundaries of seminiferous tubules and extremely few sperm within the lumen (Figure 1D).

Statistical analysis showed that the percentage of normal seminiferous tubules (Type I) was significantly decreased and that of abnormal seminiferous tubules (Type II, III, and IV) was significantly increased in PS-NPs group compared with the control group (*p* < 0.05, Figure 1E). The percentage of normal seminiferous tubules (Type I) in the APS group have no difference with the control group (*p* > 0.05, Figure 1E). There was no difference between control group and APS treatment group in the percentage of types II, III, and IV (*p* >0.05, Figure 1E).

The testosterone content was significantly lower in the PS-NPs group than the control group (*p* < 0.05, Figure 1F). The testosterone content in the APS group was no significant difference with the control group (*p* > 0.05, Figure 1F).

### 3.2 Accumulation of PS-NPs in the epididymis

**Figure 2:**
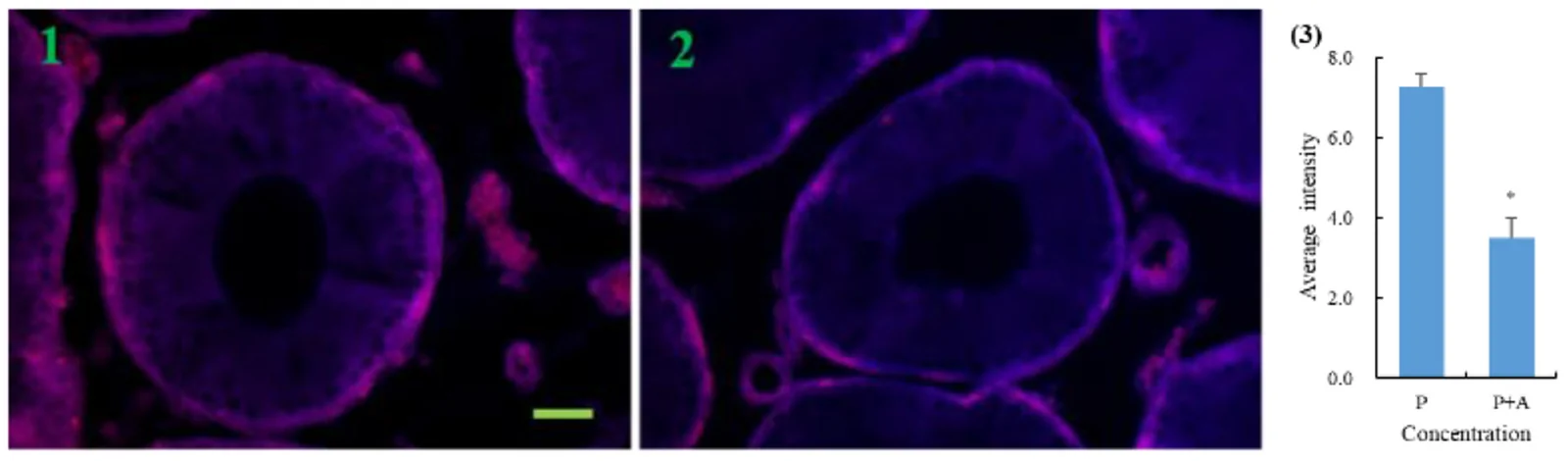
Effect of APS on the accumulation of PS-NPs in the epididymis. **(1)** Fluorescent PS-NPs at 50 mg/kg/day group; **(2)** fluorescent PS-NPs at 50 mg/kg/day and APS at 100 mg/kg/day group; Bar = 20 μm. The number of biological replicates was 6 mice.

The average intensity of PS-NPs attached to the epididymal epithelium was significantly higher in the PS-NPs group than the APS group (*p* < 0.05) (Figure 2).

### 3.3 Sperm quality, density, motility, and deformity rate

**Figure 3:**
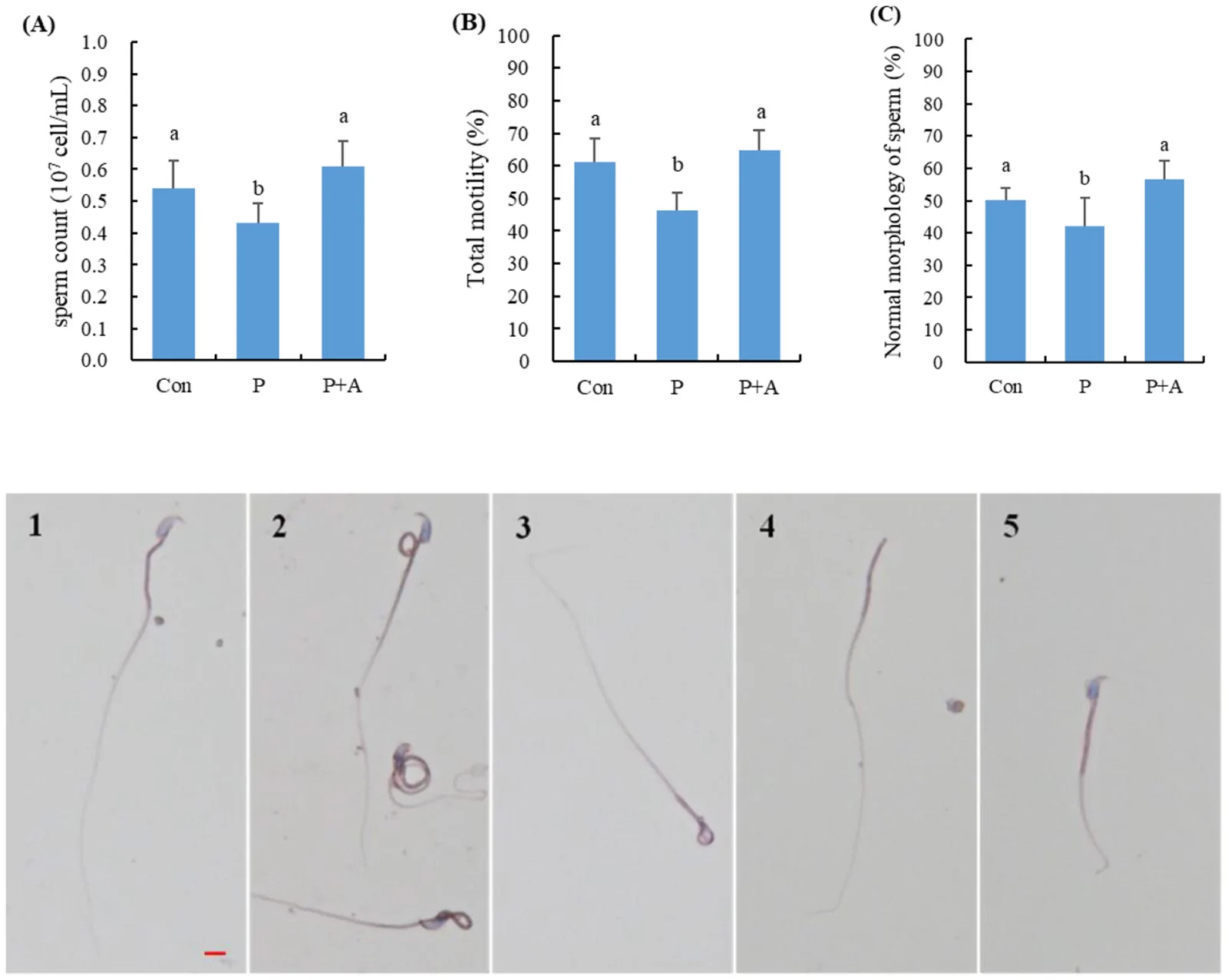
Effect of APS on sperm quantity and quality in mice exposed to PS-NPs. **(1)** Normal morphology; **(2)** Neck folding; **(3)** Acrosome shortage; **(4)** Headless; **(5)** No tail; × 400. Data are presented as the mean ± standard deviation (*n* = 6; *p < 0.05). Bar = 5 μm. The number of biological replicates was 6 mice.

The sperm density (Figure 3A), motility (Figure 3B), and normal rate (Figure 3C) were significantly lower in the PS-NPs group than the control group (*p* < 0.05). The sperm density, motility, and normal morphology rate were no significant difference in the APS group with the control group (*p* > 0.05, Figure 3).

### 3.4 PS-NPs induced oxidative stress in mice

**Figure 4:**
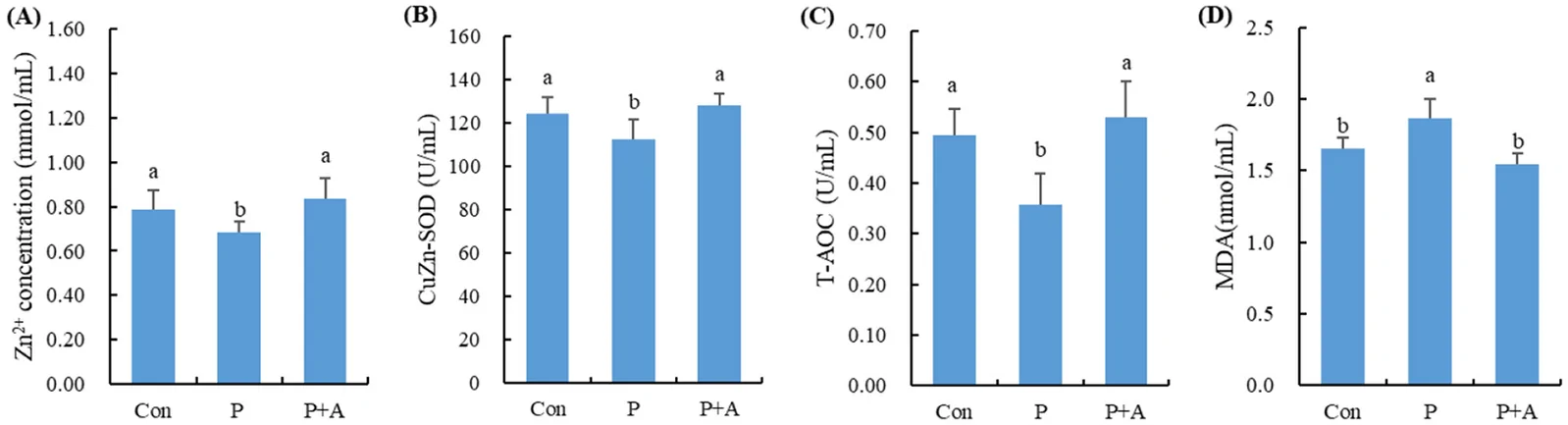
Effect of APS on oxidative stress in mice exposed to PS-NPs. Data are presented as the mean ± standard deviation (*n* = 6; *p < 0.05). The number of biological replicates was 6 mice.

The concentration of Zn^2+^ (Figure 4A), activities of CuZn-SOD (Figure 4B) and T-AOC (Figure 4C) in epididymal semen were significantly lower in the PS-NPs group than the control group (*p* < 0.05). The concentration of Zn^2+^, activities of CuZn-SOD and T-AOC in epididymal semen were significantly higher in the APS group than the PS-NPs group (*p* < 0.05), similar to the control group (*p* > 0.05).

The concentration of MDA in epididymal semen were significantly higher in the PS-NPs group than the control group (*p* < 0.05, Figure 4D). The concentration of MDA in epididymal semen were no significant difference in the APS group with the control group (*p* > 0.05).

### 3.5 Effect of exposure to PS-NPs on protein tyrosine phosphorylation

**Figure 5:**
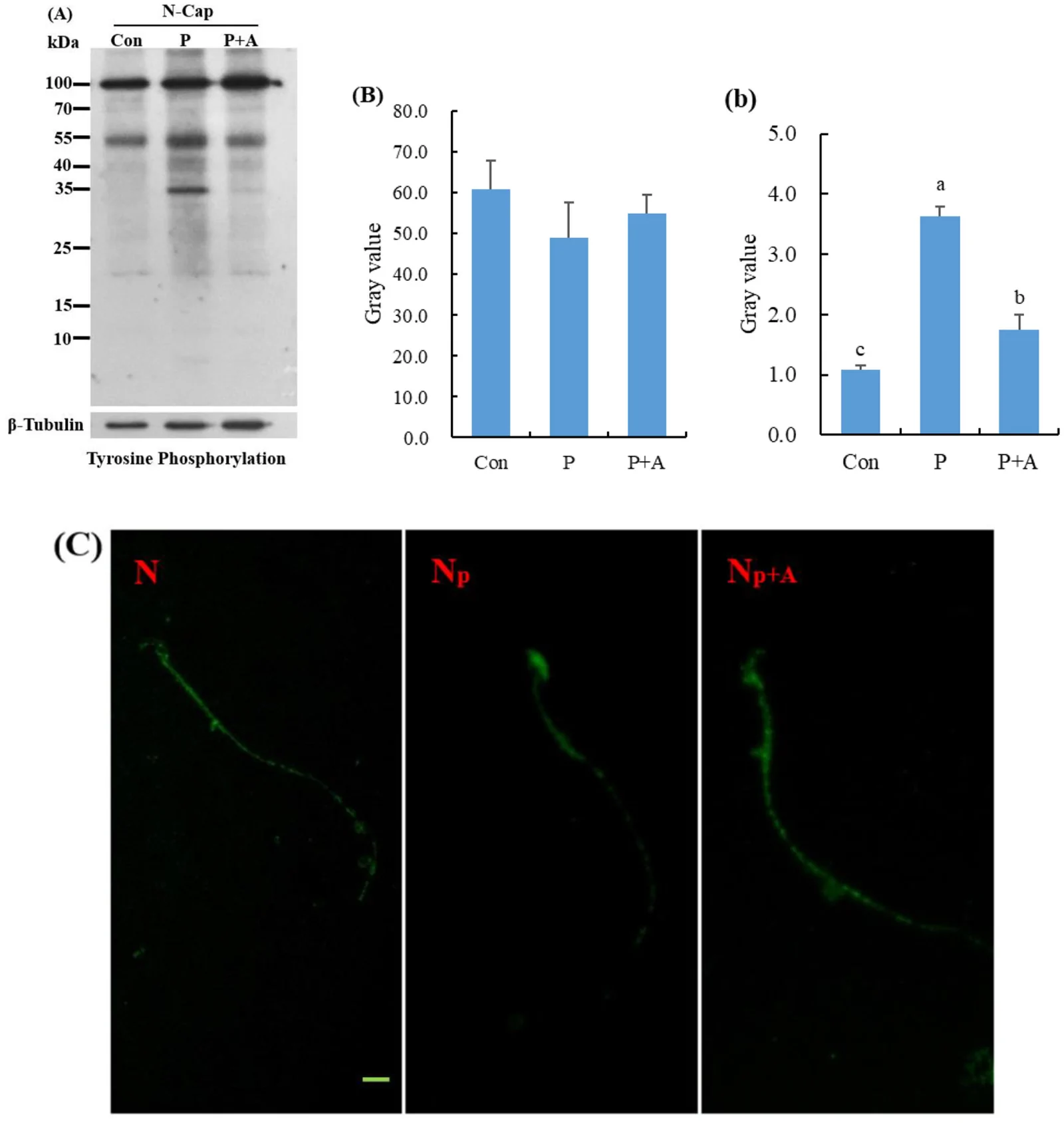
Effect of APS on tyrosine phosphorylation of non-capacitated sperm exposed to PS-NPs. **(A)** Western blot analysis was performed using an antibody against phosphotyrosine. **(B)** Non-capacitated sperm at various protein gray values of total protein tyrosine phosphorylation (*n* = 3). (b) Non-capacitated sperm at protein gray values of 32 ~ 35 KDa protein tyrosine phosphorylation (*n* = 3). β-Tubulin was used as an internal control. Different lowercase letters indicate significant differences (*p* < 0.05). **(C)** Immunolocalization of tyrosine-phosphorylated proteins in mouse sperm. Sperm cells were visualized using a fluorescence microscope ( × 400). Bar = 5 μm. N, Mouse sperm were incubated in non-capacitating medium for 90 min. The number of biological replicates was 3 mice.

Oral administration of PS-NPs induced tyrosine phosphorylation of a 32 ~ 35-kDa protein in non-capacitated sperm (Figure 5B). Protein immunofluorescence localization found that exposure to PS-NPs increased tyrosine phosphorylation in the non-capacitated sperm head, which was decreased in the non-capacitated sperm flagella (Figure 5C). Oral administration of APS alleviated the intensity of protein tyrosine phosphorylation induced by exposure to PS-NPs (Figure 5C). The protein immunofluorescence localization revealed that APS weakened the intensity of proteins tyrosine phosphorylation of non-capacitated sperm head induced by PS-NPs and enhanced proteins tyrosine phosphorylation intensity of non-capacitated sperm flagella (Figure 5C). Notably, there was no significant difference in the gray values of protein tyrosine phosphorylation among the groups (Figure 5B; *p* > 0.05).

**Figure 6:**
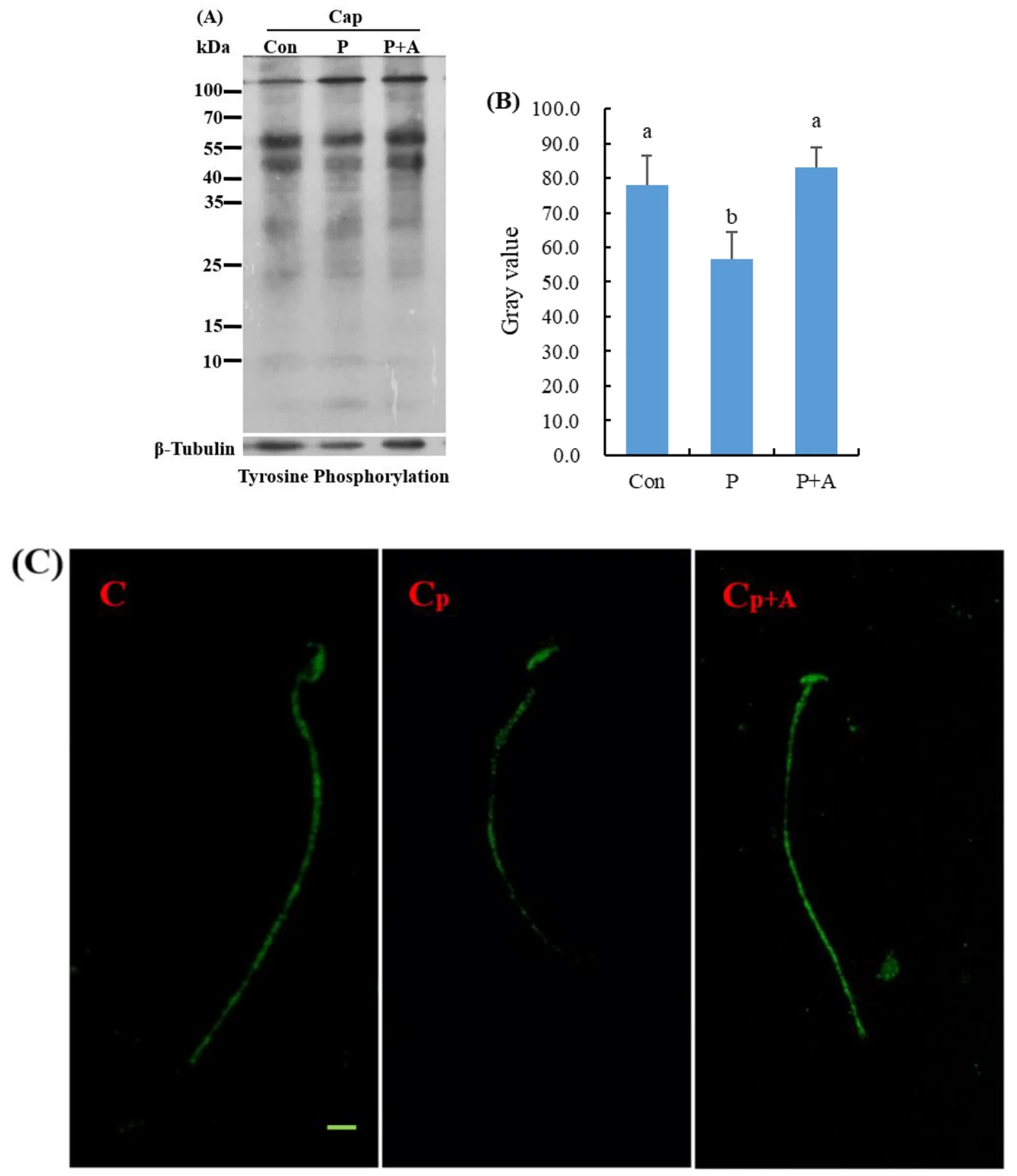
Effect of APS on tyrosine phosphorylation of capacitated sperm exposed to PS-NPs. **(A)** Western blot analysis was performed using an antibody against phosphotyrosine. **(B)** Capacitated sperm at various protein gray values of tyrosine phosphorylation (*n* = 3). β-Tubulin was used as an internal control. Different lowercase letters indicate significant differences (*p* < 0.05). **(C)** Immunolocalization of tyrosine-phosphorylated proteins in mouse sperm. Sperm cells were visualized using a fluorescence microscope ( × 400). Bar = 5 μm. C, Mouse sperm were incubated in capacitating medium for 90 min. The number of biological replicates was 3 mice.

After capacitated incubation, the gray value of total protein tyrosine phosphorylation in PS-NPs group was significantly lower than in the control group (*p* < 0.05, Figure 6B). This protein was mainly localized in the capacitated sperm flagella. Protein immunofluorescence localization showed that the fluorescence intensity of capacitated sperm tails exposed to PS-NPs was decreased (Figure 6C). The capacitated sperm of protein tyrosine phosphorylation on APS group was no significant difference with the control group (*p* > 0.05, Figure 6B). Fluorescence localization showed that PS-NPs had a significant impact on protein tyrosine phosphorylation in the middle- and principal-pieces of the capacitated sperm flagellum (Figure 6C).

## 4 Discussion

PS-NPs pose potential hazards to the reproductive systems of mammals (10). Given the widespread application of plastics in production and daily life, these hazards cannot be eliminated in the short term. Therefore, selecting substances with mitigating effects is crucial to minimize potential harm.

Androgens play a crucial role in the maturation and function of the testes and epididymis (13). In this experiment, PS-NPs caused loose arrangement of germ cells and lumens in the seminiferous tubules of the testes, leading to a greater proportion of deformed sperm, characterized by neck folding, acrosome loss, head loss, and shortening of the flagellum. The experimental results showed that exposure to PS-NPs reduced levels of testosterone, which plays an important role in sperm maturation in the epididymis and may directly affect sperm motility. After treatment with APS, the testicular pathological injury, sperm count, sperm density, and the proportion of morphologically normal sperm in mice were markedly ameliorated, demonstrating that testicular injury induced by PS-NPs can be alleviated by APS intervention.

Oral administration of PS-NPs triggers structural developmental defects in the sperm acrosome of mice. Acrosomal injury may serve as a key mechanism underlying the decline in male fertility induced by PS-NPs. Consistent with this notion, the present study verified that PS-NPs exposure resulted in multiple abnormalities in epididymal sperm, including acrosomal shedding. During spermatogenesis in the testis, APS could effectively suppress the development of abnormal sperm in testicular tissues, mitigate damage to testicular cells, and maintain the structural integrity of sperm—an essential prerequisite for normal sperm function.

The epididymis is a critical organ responsible for post-testicular sperm maturation (14). Testicular spermatozoa are inherently immotile under physiological circumstances and acquire progressive motility upon entering the epididymis, where they are continuously exposed to epididymal secretions (15). Fluorescently tagged PS-NPs adhere to the epididymal epithelium and trigger damage to epithelial cells. Such cellular injury compromises both the sperm transport capability and secretory activity of the epididymal epithelium (14). Notably, APS intervention effectively mitigates the deposition of PS-NPs in the epididymal epithelial layer.

Zinc exerts vital regulatory effects on testosterone bioactivity (16). Enriched in testicular tissues, this trace element is indispensable for reproductive processes and acts as an essential cofactor participating in spermatogenesis (17, 18). Moreover, zinc constitutes an integral structural subunit of CuZn-SOD (14). Cumulative evidence has demonstrated that the toxic effects of PS-NPs are tightly associated with the overproduction of ROS. Excessively accumulated ROS can trigger mitochondrial dysfunction and DNA oxidative damage (6). In the present study, PS-NPs exposure reduced zinc ion concentrations and CuZn-SOD activity in seminal plasma. This disruption sequentially lowered T-AOC and elevated MDA levels, establishing oxidative imbalance within the sperm microenvironment. In contrast, APS administration markedly reversed these adverse oxidative alterations and restored the physiological homeostasis of the sperm microenvironment.

Following testicular development, mammalian sperm barely synthesize endogenous proteins *de novo*. Apart from translational modifications of intrinsic sperm proteins, spermatozoa released from the testis remain largely transcriptionally and translationally quiescent (19). Reversible phosphorylation and dephosphorylation of proteins serve as the predominant molecular mechanism governing signal transduction, enzymatic activity regulation and functional modulation in mammalian sperm. Such post-translational modifications are also critical for sperm-egg recognition and the successful accomplishment of fertilization (20).

After culturing of epididymal sperm in non-capacitating medium, analysis of relevant indicators showed that exposure to PS-NPs caused a significant increase in tyrosine phosphorylation of a 32 ~ 35 KDa protein localized to the acrosome, which may be a 32 kDa acrosome matrix protein (21). Administration of PS-NPs cause abnormal tyrosine phosphorylation of non-capacitated sperm proteins, which may lead to “capacitation-like” of non-capacitated sperm. In clinical practice, infertile men often experience premature acrosome activation, which can be caused by abnormal responses to calcium ions, spontaneous shedding of the acrosome and release of acrosome contents (22). APS can eliminate tyrosine phosphorylation of non-capacitated sperm proteins induced by PS-NPs, thereby protecting the acrosome response of sperm exposed to PS-NPs.

After capacitated incubation epididymal sperm *in vitro*, the relevant indicators showed that exposure to PS-NPs caused increased tyrosine phosphorylation of a 120 KDa capacitated sperm protein, with fluorescence localized to the principal-pieces of the flagella, but yet caused a decrease in tyrosine phosphorylation of a 45 KDa protein, which localized to the principal-pieces of the flagella. Previous studies have shown that exposure to PS-NPs can inhibit acidification of the rat epididymal lumens, reduce sperm motility (23). It is speculated that abnormal protein tyrosine phosphorylation may be responsible for the significant impact of PS-NPs on reduced capacitated sperm motility. A tyrosine phosphorylated protein with a molecular weight of around 45 KDa was found in mouse capacitated sperm, with representative proteins, such as ODF2, located in the outer dense fibers of the sperm flagellum. The tyrosine phosphorylated protein located at the flagella plays a role in structural support, movement regulation, and signal integration functions (24). The damage caused by PS-NPs to tyrosine phosphorylation of capacitated sperm proteins may affect sperm motility and energy production, thereby affecting fertilization capacity. APS can effectively alleviate the effect of PS-NPs on protein tyrosine phosphorylation in capacitated sperm.

Despite these findings, it is important to acknowledge certain limitations of the present study. The key consideration is that absence of APS-only control group, which limits the possibility to distinguish between APS's independent biological effects and its protective effects against PS-NPs. Meanwhile, exposure duration to the treatments limited to 10 days, which is insufficient for a full spermatogenic cycle (~35 days in mice).

## 5 Conclusion

PS-NPs may cause abnormalities to the mouse testicular structure by accompanied by ROS accumulation, resulting in a decrease in number, normal morphology, and vitality of sperm. The effects of PS-NPs on the testes and epididymis are comprehensively reflected in the significant decrease in serum testosterone levels, the increase in zinc ion concentration and corresponding oxidative indicators, and the decreased accumulation of ROS and activities of antioxidant enzymes. The impact of PS-NPs on the male reproductive system of mice is comprehensive, affecting sperm growth, quality, and function in multiple ways. The accumulation of PS-NPs in the epididymal epithelium affects the maturation of sperm, leading to abnormal protein tyrosine phosphorylation in epididymal sperm. PS-NPs can induce a significant increase in protein tyrosine phosphorylation of a 32 ~ 35-kDa protein of non-capacitated mouse sperm, which may lead to premature acrosome reactions, similar to the “capacitation-like” phenomenon. In capacitated sperm, protein tyrosine phosphorylation of the principal-pieces flagellum is decreased.

This study expanded prior analyses of indicators of mature capacitated sperm exposed to PS-NPs and explored the protective effects of APS on testicular and epididymal structure and function in response to exposure to PS-NPs. APS have a relieving effect on oxidative damage to sperm caused by exposure to PS-NPs by enhancing antioxidant capacity, providing a suitable physicochemical environment, regulating protein tyrosine phosphorylation modification, and improving capacitation parameters. Therefore, it is speculated that APS can alleviate the harm caused to sperm by environmental pollutants.

The impact of NPs on reproductive health is a critical issue that extends beyond individual organisms, affecting both public health and animal welfare. This study highlights the potential risks associated with exposure to NPs, particularly in relation to reproductive functionality. However, several limitations should be acknowledged, such as sample size, methodology, and scope. To address these issues, Subsequent research will appropriately expand the animal sample size, supplement the APS group, and set up multiple gradient drug concentrations to improve the reliability and completeness of the study.

## Data Availability

The raw data supporting the conclusions of this article will be made available by the authors, without undue reservation.
